# Conversion of patellofemoral arthroplasty to total knee arthroplasty: A matched case-control study of 13 patients

**DOI:** 10.1080/17453670902805031

**Published:** 2009-02-01

**Authors:** Hans-Peter W van Jonbergen, Dirk M Werkman, Albert van Kampen

**Affiliations:** ^1^Department of Orthopedic Surgery, Deventer HospitalDeventerthe Netherlands; ^2^Department of Orthopedic Surgery and Orthopedic Research Laboratory, University Medical CenterSt. Radboud, Nijmegenthe Netherlands

## Abstract

**Background and purpose** The long-term outcome of patellofemoral arthroplasty is related to progression of femorotibial osteoarthritis with need for conversion to total knee arthroplasty. We investigated whether prior patellofemoral arthroplasty compromises the results of total knee arthroplasty.

**Methods** 13 patients who had had 14 Richards type II patellofemoral arthroplasties converted to total knee arthroplasty because of femorotibial osteoarthritis, were individually matched to a control group of 13 patients with 14 primary total knee arthroplasties. The mean follow-up times for the patients and the control group were 5.7 (2–13) years and 5.2 (2–13) years, respectively. Clinical outcome was assessed using Knee Society score (KSS), WOMAC score, range of motion, and complications.

**Results** KSS and WOMAC scores were similar in the two groups (KSS in patient and control groups: 82 and 86 (p = 0.6); KSS function: 76 and 88 (p = 0.5); WOMAC score: 33 and 21 (p = 0.1)). Within 6 months after conversion, 3 knees had to be manipulated under anesthesia for limited motion. No patients in the control group required manipulation under anesthesia.

**Interpretation** Patellofemoral arthroplasty appears not to have a negative effect on the outcome of later total knee arthroplasty.

## Introduction

Patellofemoral arthroplasty is a treatment alternative for isolated patellofemoral osteoarthritis. The long-term outcome is related to malposition of the prosthesis, the progression or development of femorotibial osteoarthritis, and—to a lesser extent—wear and/or loosening of the patellar component. Several reports have described the progression of symptomatic femorotibial osteoarthritis as an important reason for conversion to total knee arthroplasty, with an overall revision rate of between 4% and 28% (Argenson et al. 2005, Leadbetter et al. 2005, Nicol et al. 2006, Ackroyd et al. 2007).

With the renewed interest in patellofemoral arthroplasty, more conversion to total knee arthroplasty due to progression of femorotibial osteoarthritis may be anticipated. Only one paper has reviewed the results of revision of a failed patellofemoral arthroplasty to a total knee arthroplasty (Lonner et al. 2006). No technical difficulties were observed, and clinical outcome as assessed by the Knee Society score (KSS) improved after revision. However, whether or not these results compare favorably with the results obtained after primary total knee arthroplasty is unknown.

We therefore performed a retrospective case-control study to compare the outcome of patients with a patellofemoral arthroplasty converted to a total knee arthroplasty with that of a matched group of patients with a primary total knee arthroplasty for femorotibial osteoarthritis.

## Patients and methods

### Patient selection

Patellofemoral arthroplasty has been performed at our institution since 1976. The entire cohort of 172 patients with 196 patellofemoral arthroplasties had a regular follow-up with clinical and radiographic examinations every 1 or 2 years.

Between October 1987 and March 2007, 23 Richards type II patellofemoral arthroplasties (Smith and Nephew, Memphis, TN) were revised to total knee arthroplasty in 22 patients (17 women) because of development of painful femorotibial osteoarthritis. No conversions had been done before 1987. 7 patients had died since conversion (of causes unrelated to surgery), and only patients with at least 2 years of follow-up were included. The index group thus consisted of 14 revision total knee arthroplasties in 13 patients (10 women), with revision surgery performed between 1993 and 2005. The study was performed with retrospective data collection and review. The study protocol was approved by the institutional Review Board (NL22632.075.0818, March 2008). A control group of 14 primary total knee arthroplasties in 13 patients was selected from the cohort of primary total knee arthroplasties performed at our institution during the same time period. The underlying diagnosis was primary osteoarthritis in all patients. Only patients with at least 2 years of follow-up were included.

To prevent cohort disparity, each case was individually matched on the basis of 7 attributes: sex, age at time of total knee arthroplasty (± 5 years), date of surgery (± 1 year), type of total knee prosthesis, duration of follow-up (± 1 year), body mass index (± 2), and radiographic grade of osteoarthritis (Table 1). No matches were made for type and number of previous procedures. The matching process was performed blind to the clinical outcome.

**Table 1.** Demographic and radiographic data for 14 knees with a patellofemoral arthroplasty prior to conversion to total knee arthroplasty (index group) and 14 knees with primary total knee arthroplasties (control group)

	Index	Control	p-value
Sex (female : male)	10 : 3	10 : 3	
No. of knees	14	14	
Age at time of total knee arthroplasty, years (range)	67 (50–77)	68 (51–76)	0.7
Follow-up, years (range)	5.7 (2.0–13)	5.2 (2.1–13)	0.1
Body mass index (range)	29 (22–35)	29 (23–34)	0.4
Kellgren grade			0.3
1	0	0	
2	6	8	
3	6	2	
4	2	4	
Ahlbäck grade			0.6
1	11	12	
2	2	0	
3	1	2	
4	0	0	

Informed consent was obtained from all patients.

### Clinical evaluation

Patients in both groups had regular follow-up with clinical and radiographic examinations every 1 or 2 years after surgery. All patients completed the Dutch version of the WOMAC 3.1 Osteoarthritis Index, range of motion was registered, and the KSS was used for outcome assessment (Insall et al. 1989).

### Radiographic evaluation

Preoperative radiographs were assessed by a radiologist for femorotibial osteoarthritis using the Kellgren and the Ahlbäck grading systems (Kellgren and Lawrence 1957, Ahlbäck 1968). Immediate postoperative radiographs (anteroposterior and lateral non-weight bearing) were evaluated to assess the position of the prosthesis. During follow-up, the radiographic examination consisted of 2 radiographs (anteroposterior standing and lateral non-weight bearing), and all sequential radiographs were assessed by a radiologist to determine loosening or wear of the prosthesis.

### Statistics

No power analysis was performed prior to this study, as all patients who had a conversion from patellofemoral to total knee arthroplasty at our institution were included. The Fisher exact probability test was used for categorical data, and the Wilcoxon signed ranks test was used to investigate differences in continuous data between groups. All p-values less than 0.05 were considered significant.

## Results

### Matching

No statistically significant differences with respect to age, preoperative grade of osteoarthritis, duration of follow-up, or body mass index were found between the two groups (Table 1).

### Previous patellofemoral arthroplasty

The mean age at patellofemoral arthroplasty was 56 (35–72) years. The Richards type II patellofemoral prosthesis was used in all patients. Further surgery after patellofemoral arthroplasty was performed in 9 knees and included 16 procedures (2 knees were manipulated under anesthesia; arthrotomy for painful bony impingement or persistent pain was done in 3 knees; 9 arthroscopies were performed (femorotibial debridement, meniscectomy, diagnostic); and 1 knee had a proximal tibial osteotomy with subsequent hardware removal). The patellofemoral prostheses had been in place before conversion to total knee arthroplasty for an average of 11 (1.2–27) years.

### Surgical procedure

In both groups, total knee arthroplasty was performed by several surgeons with similar experience. Before 1999, the posterior-stabilized Insall-Burstein total knee prosthesis was used (Insall-Burstein; Zimmer, Warsaw, IN), and from 1999 onwards a NexGen posterior-stabilized total knee prosthesis was used (NexGen; Zimmer). In each group, 3 Insall-Burstein prostheses and 11 NexGen prostheses were used. Operative records were available for all patients.

**Figure 1.**
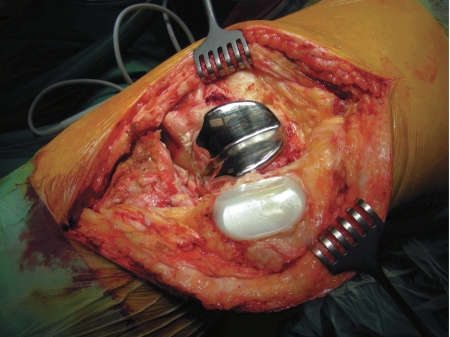
Patellofemoral prosthesis in situ.

**Figure 2.**
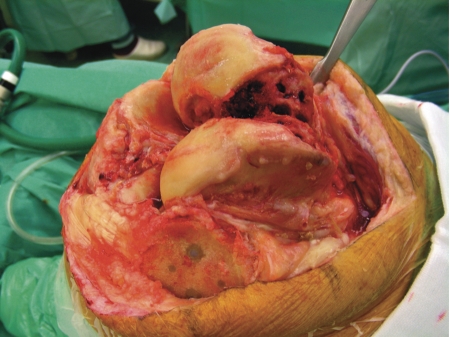
After removal of femoral and patellar components.

**Figure 3.**
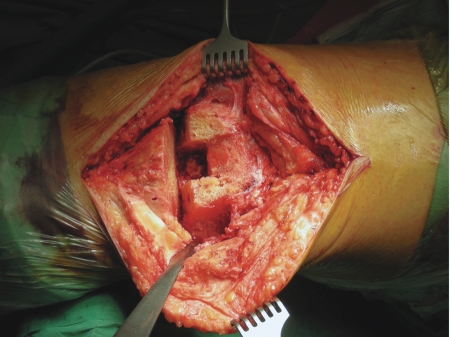
After preparation of distal femur and proximal tibia using the standard cutting blocks.

At conversion, both the femoral and patellar component were removed in all cases (Figures 1 and 2). The distal femur was prepared using standard cutting blocks with resection of the soft cancellous bone directly beneath the femoral component (Figure 3). Patellar thickness was restored using the standard patellar component for total knee arthroplasty. After preparation of the proximal tibia and insertion of trial components, patellofemoral stability was tested through a full range of motion before the definitive components were cemented in place. Condylar support for the femoral component was adequate in all cases; no additional metal augmentation was required for any of the knees. Identical surgical procedures and cutting blocks were used for primary total knee arthroplasty in the control group. In all cases, patellar resurfacing was performed using the standard patellar component for total knee arthroplasty.

Radiographs taken immediately postoperatively showed adequate positioning of the prosthesis in all 28 knees. Patients were allowed immediate protected weight bearing with crutches. All patients routinely received coumarine prophylactically for 8 weeks. Data for operative time and blood loss were incomplete and were therefore not included in the analysis.

### Complications and further surgery

Within 6 months of conversion from patellofemoral arthroplasty to total knee arthroplasty, 3 knees (in 3 patients) had to be manipulated under anesthesia for failure to achieve 90 degrees of flexion by 6 weeks postoperatively. In 2 of these patients, manipulation had also been necessary after the previous patellofemoral arthroplasty. For the 3 patients requiring manipulation, the mean time between patellofemoral arthroplasty and conversion was 15 (13–17) years. The patients had had an average of 4 previous knee operations before conversion and achieved a mean preoperative flexion of 98 (85–120) degrees.

One patient (with 3 previous procedures before conversion, including patellofemoral arthroplasty, proximal tibial osteotomy, and subsequent hardware removal) had signs of infection of the prosthesis within this time period, and received adequate operative and antibiotic treatment. To date, he has no clinical signs of infection of the total knee prosthesis. No patients had patellofemoral-related complications.

In the control group, no further surgery or manipulation under anesthesia within this time period was required or performed, and no complications were observed.

### Clinical outcome

The functional outcome using KSS, WOMAC scores, and range of motion were similar in both groups (Table 2). Additional analysis of subscores of the KSS (pain, range of motion, and stability) and WOMAC (pain, stiffness, and function) showed no statistically significant differences between the index and control groups. Preoperative KSS and WOMAC scores were not available for the entire group, so comparison of improvement between the groups was not possible.

**Table 2.** Clinical outcome after total knee arthroplasty. Values are mean (SD)

	Index	Control	p-value
KSS (max. 100)	82 (19)	86 (10)	0.6
KSS function (max. 100)	76 (31)	88 (10)	0.5
WOMAC (max. 96)	33 (23)	21 (16)	0.1
Preoperative flexion (degrees)	108 (14)	110 (12)	0.7
Postoperative flexion (degrees)	117 (13)	116 (11)	0.9

### Radiographic outcome

At final follow-up, none of the knees showed signs of radiographic loosening and/or wear.

## Discussion

Our findings suggest that patellofemoral arthroplasty has no negative effect on the outcome of later total knee arthroplasty.

Although no statistically significant differences in KSS and WOMAC scores between the groups were found, the high number of manipulations in the patellofemoral conversion group may be an important observation. Recently, Lonner et al. (2006) assessed the conversion of patellofemoral knee replacement to total knee arthroplasty in 12 patients and found that 2 of the patients required manipulation under anesthesia 6 weeks after conversion. In our complete cohort of patients with primary patellofemoral arthroplasty, 12 of 196 knees (6%) in 11 of 172 patients had to be manipulated under anesthesia within 6 months of arthroplasty. Several other studies have noted a need for manipulation under anesthesia for stiffness within 6 months of patellofemoral arthroplasty, with reported incidences ranging from 3% to 14% (Arciero and Toomey 1988, Argenson et al. 1995, 2005, de Winter et al. 2001, Ackroyd and Chir 2005). The reasons underlying the need for manipulation may be complex and possibly related to the primary patellofemoral disease process and surgical treatment before patellofemoral arthroplasty. The reported prevalence of stiffness after primary total knee arthroplasty varies from 1% to 5%, although it is notable that a commonly used definition of stiffness following knee arthroplasty is lacking (Kim et al. 2004, Yercan et al. 2006, Keating et al. 2007). A history of previous knee surgery and the preoperative range of motion are important predictors of the range of motion after total knee arthroplasty.

Our study has several limitations. Although progression or development of femorotibial osteoarthritis is an important reason for conversion to total knee arthroplasty, large populations need to be tracked for long periods of time to observe disease development. Also, follow-up after conversion to total knee arthroplasty should be extended to several years to reliably evaluate the results of conversion. Thus, a case-control study was designed using a cohort of patients with conversion to total knee arthroplasty. With the small number of patients available in our study, no statistically significant differences in clinical outcome using KSS and WOMAC scores were found. Furthermore, more discriminatory knee scoring systems may be necessary (Paxton and Fithian 2005). Potential differences in improvement between the two groups were not evaluated, as preoperative KSS and WOMAC scores for the entire group were not available.

To date, only 1 paper has reported the results of revision of failed patellofemoral arthroplasty to a total knee arthroplasty (Lonner et al. 2006). Conversion of patellofemoral arthroplasty to a NexGen Legacy posterior-stabilized total knee arthroplasty was performed in 12 patients for patellar maltracking or degenerative joint disease. At a mean follow-up of 3 (2–5) years, all patients had higher Knee Society clinical and functional scores. No technical difficulties were encountered during revision. No patellar components were revised, since the femoral component was accommodating to the original dome-shaped all-polyethylene patellar components of the Lubinus, Autocentric, Low-Contact Stress or Avon patellofemoral prostheses. At our institution, however, the patellar component was revised in all cases. The Richards type II all-polyethylene patellar prosthesis has a long midline central ridge (Figure 1). Retaining the patellar prosthesis could have resulted in maltracking or increased wear of the polyethylene; thus, some authors have suggested that patellofemoral arthroplasty should use a universal patellar component that is compatible with total knee systems, thus obviating the need for revision of the patella (Argenson et al. 2005).

We did not experience technical problems during conversion. Removal of the trochlear component proved to be straightforward, without any substantial loss of bone. Use of the standard total knee replacement cutting blocks resulted in an optimally prepared distal femur, and therefore metal augmentation was not required in any of the patients. This was also observed by Lonner et al. (2006), who noted that condylar support in each knee was uncompromised.
